# The Role of Stabilin-1 in Lymphocyte Trafficking and Macrophage Scavenging in the Liver Microenvironment

**DOI:** 10.3390/biom9070283

**Published:** 2019-07-16

**Authors:** Daniel A. Patten, Shishir Shetty

**Affiliations:** 1 Centre for Liver and Gastrointestinal Research, Institute of Immunology and Immunotherapy, Medical School, University of Birmingham, Edgbaston, Birmingham B15 2TT, UK; 2 NIHR Birmingham Biomedical Research Centre, University Hospitals Birmingham NHS Foundation Trust and University of Birmingham, Birmingham B15 2TT, UK

**Keywords:** Liver, Inflammation, Fibrosis, Stabilin-1

## Abstract

Chronic liver diseases are a major global health burden, and cases of these conditions continue to rise in many countries. A diverse range of insults can lead to chronic liver disease, but they are all characterised by the infiltration and accumulation of immune cells within liver tissue and, if progressive, can lead to tissue fibrosis and cirrhosis. In this review, we focus on the role of stabilin-1 in two key processes that contribute to liver disease, namely, the recruitment of lymphocytes into liver tissue and the response of macrophages to tissue injury. Stabilin-1 is constitutively expressed on the sinusoidal endothelium of the liver and contributes to the homeostatic scavenging function of these cells. Epithelial damage in the context of chronic liver disease leads to the upregulation of stabilin-1 at sites of tissue injury, specifically at sites of immune cell recruitment and on subpopulations of hepatic macrophages. Functionally, stabilin-1 has been shown to mediate transendothelial migration of lymphocyte subsets in the setting of pro-inflammatory-activated human liver endothelium. In experimental models of liver fibrosis, stabilin-1 promotes the uptake of products of chronic oxidative stress by a subset of hepatic macrophages and suppresses their release of pro-inflammatory mediators that regulate tissue remodelling. These studies highlight the active contribution that scavenger receptors such as stabilin-1 can make in regulating chronic inflammation and tissue fibrosis, and their potential as novel therapeutic targets for these conditions.

## 1. Introduction

Concurrent with the widespread obesity epidemic and increasing alcohol consumption worldwide, chronic inflammatory liver diseases in adults are significantly contributing towards a global burden on human health; indeed, the incidence of liver disease in the UK alone has risen over 400 % since the 1970s to become the third most common cause of premature death [1]. In addition to the increasing incidence of non-alcoholic steatohepatitis (NASH; fatty liver disease) and alcohol-related liver disease (ARLD) [2], other aetiologies, such as viral hepatitis and autoimmune liver diseases, are also contributing to the rising occurrence of chronic liver disease [3,4]. Each disease aetiology elicits a specific pattern of injury, which is largely dependent on the site of initial damage; for example, NASH is triggered by lipotoxicity in hepatocytes, resulting in parenchymal inflammation [5], whereas autoimmune disease, primary sclerosing cholangitis (PSC), is driven by bile duct injury and characterised by portal inflammation, ductular proliferation and loss of bile duct function [6]. Nevertheless, regardless of the aetiology, patients with progressive disease follow a common pathophysiology, underpinned by excessive immune cell infiltration of liver tissues, immune activation and fibrosis. Fibrosis, or scarring, results from the activation of hepatic stellate cells, a population of liver-resident pericytes, which differentiate under inflammatory conditions to a myofibroblast phenotype, resulting in the production of extracellular matrix proteins [7]. In chronic liver diseases, sustained activation of hepatic stellate cells, perpetuated by the chronicity of the inflammatory insult, leads to the excessive accumulation of scar tissue within the liver, which ultimately culminates in loss of liver function, cirrhosis and, eventually, end-stage liver failure or hepatocellular cancer (HCC).

## 2. Recruitment of Immune Cells to the Liver

**Figure 1:**
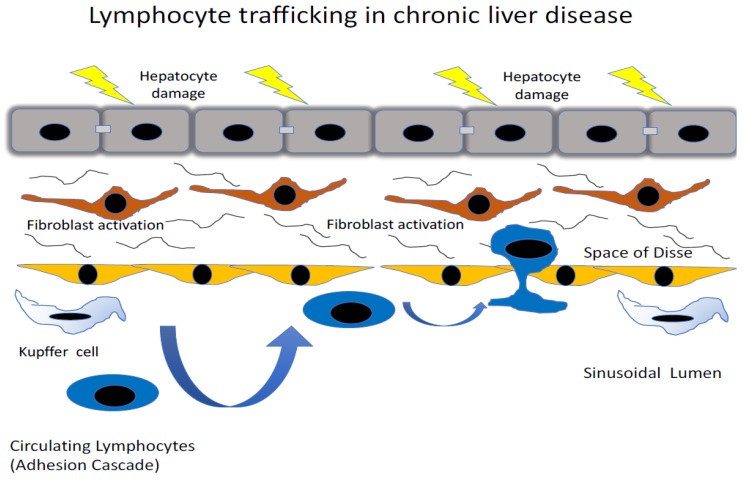
Lymphocyte trafficking in chronic liver disease. Lymphocyte recruitment from the circulation into liver tissue occurs within the low shear specialized channels of the hepatic sinusoids that are lined by hepatic sinusoidal endothelial cells and the macrophage liver resident population, the Kupffer cells. During liver injury, the signals of damaged epithelial cells lead to activation of hepatic stellate cells in the Space of Disse underneath the sinusoidal endothelium. The sinusoidal endothelium itself is activated and upregulates the expression of adhesion molecules, which promote the recruitment of lymphocytes from circulation in an organ specific manner. This involves an adhesion cascade where lymphocytes in circulation undergo a tethering step leading to firm adhesion and activation on the endothelial surface, followed by their transendothelial migraton into liver tissue.

In the event of injury or infection, such as those repeatedly incurred in chronic liver diseases, immune cells are recruited from the systemic circulation of the blood into the inflamed tissue, in order to eliminate the inflammatory trigger and/or contribute to tissue repair [8]. This migration of immune cells from the blood is mediated via a multi-step process, collectively known as the leukocyte adhesion cascade (Figure 1). During the initial stages of the leukocyte adhesion cascade, immune cells are captured from the flow of circulating blood and roll on the luminal surface of the blood vessel. Subsequently, immune cells undergo arrest, followed by firm adhesion and, finally, they transmigrate, either between (paracellular pathway) or through (transcellular pathway), the cells of the endothelial barrier and into the tissue [9]. This sequential process is orchestrated by a large number of endothelial-expressed chemokines [10] and adhesion molecules [8,11], which are able to elegantly control the subset of immune cell recruited to the site of inflammation in a highly specific manner. Additionally, due to its potential to disrupt endothelial integrity, the process of transmigration is itself a highly selective process as it requires extensive cytoskeletal remodelling to accommodate the passage of the immune cell [12]. Unsurprisingly, under physiological conditions, the transmigration of leukocytes is stringently regulated by the endothelial cell to minimise both vascular leakage and the number of immune cells crossing the vascular wall; nevertheless, this process can become highly dysregulated in the diseased state. More recently, it has become clear that leukcocytes can migrate across endothelium through two distinct pathways: a conventional paracellular route via cellular junctions, and a second route termed transcellular migration where a leucocyte migrates directly through the endothelial cell body. Why leucocytes may use two distinct pathways and the functional consequences are unclear, but the act of leukocyte migration also acts to ‘prime’ the tissue-infiltrating immune cells in order to produce an efficient and effective immunological response in the relevant inflamed tissue [13]; however, this process can also become imbalanced and further perpetuate the disease state.

**Figure 2:**
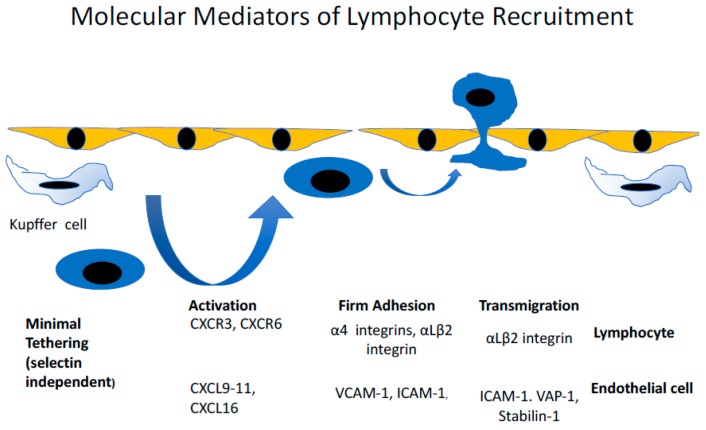
The molecular mechanisms of lymphocyte trafficking in liver disease. The recruitment of lymphocytes in the hepatic sinusoids is mediated by a combination of surface receptors and chemoattractant cytokines. In conventional recruitment, lymphocytes undergo a rolling step that is mediated by selectins. Selectins are absent in the sinusoidal channels, and after a brief tethering, lymphocytes undergo activation and firm adhesion mediated by a combination of chemokines presented on the endothelium and binding to chemokine receptors on the lymphocyte surface (for example, the inflammatory chemokines CXCL9, 10, and 11 binding to the chemokine receptor CXCR3, or the chemokine CXCL16 binding to CXCR6). Following activation, the lymphocytes bind via integrins to intercellular adhesion molecule-1 (ICAM-1) and vascular cell adhesion molecule-1 (VCAM-1). The final step is transendothelial migration, which has been shown to be mediated by ICAM-1, vascular adhesion protein-1 (VAP-1), and stabilin-1.

**Figure 3:**
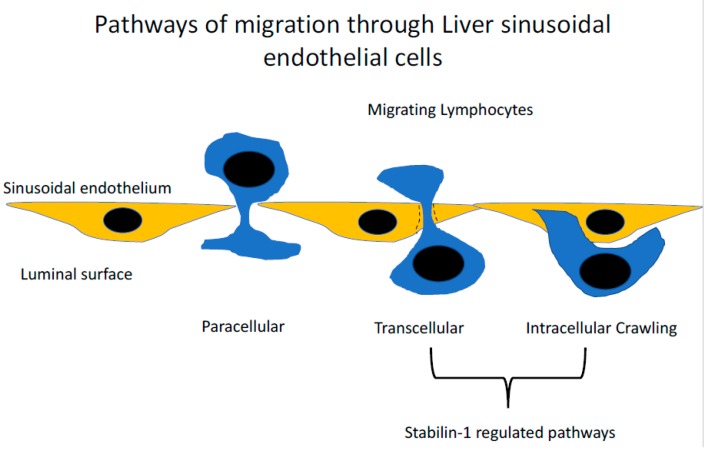
The routes taken by lymphocytes during transendothelial migration. Detailed analysis of the last step of the adhesion cascade where lymphocytes cross the endothelial barrier have demonstrated that several routes can be taken. The conventional route is the paracellular route, where lymphocytes migrate directly between endothelial cellular junctions. The second route, which appears to occur in the liver at a high frequency, is the migration of lymphocytes directly through the body of the cell, termed the transcellular migration. This route of migration has been described in other microvascular beds including the lymphatics and bone marrow. An additional novel route has also been described where lymphocytes invade into the body of the endothelial cell and then migrate directly into the adjacent endothelial cell termed ‘intracellular crawling’. Stabilin-1 has been shown to contribute to both transcellular migration and intracellular crawling.

Generally, immune cell recruitment occurs in the post-capillary venules of the relevant inflamed tissues; however, in the liver, this process occurs within the unique low shear flow environment of the narrow hepatic sinusoidal microvasculture [14]. The hepatic sinusoids are lined by a highly specialised and functionally unique endothelium [15]. Liver sinusoidal endothelial cells (LSEC) are phenotypically very distinct from conventional vascular endothelial cells and are more analogous to lymphatic endothelial cells [16]; they also lack a basement membrane [17], with atypical cellular junctions [18,19] and membranous pores, called fenestrations [20]. The combinatory effect of the structural characteristics of the sinusoids and the phenotype of the LSEC themselves significantly modifies the mechanism of immune recruitment in the liver. The narrow, low shear stress environment of the hepatic sinusoids negates the initial rolling steps of the leukocyte adhesion cascade [14]. As a consequence, LSECs express negligible levels of classical adhesion molecules, such as selectins [16]. The selectin family of proteins are key in the initial stages of the leukocyte adhesion cascade in more conventional endothelial cells [21], and their lack of expression in LSEC presents the opportunity for a range of atypical adhesion molecules to play a more prominent role in the recruitment process [22,23,24,25] (Figure 2). In addition, the latter stages of the adhesion cascade are also affected by the phenotype of LSEC, with studies utilising primary human LSEC in vitro demonstrating that a significant proportion (~40%) of adhered lymphocytes preferentially migrated via the transcellular route, a process that was significantly reduced in more conventional endothelial cells (human umbilical vein endothelial cells; HUVEC) [19]. The same study also highlighted a novel migratory pathway in which lymphocytes were able to migrate horizontally in the endothelial layer into adjacent LSEC. These processes were interferon-γ-mediated and were thought to be facilitated by the unique junctional complexes expressed by LSECs (Figure 3).

As is appropriate to their primary physiological function in the removal of endogenous and exogenous waste from the bloodstream [26], LSEC are known to express a wide array of scavenger receptors (SRs) [15,27]. There is increasing evidence that some of these endothelial-expressed scavenger receptors exhibit a secondary function as atypical adhesion molecules, as they are able to directly bind leukocyte-expressed ligands, thus facilitating the trafficking of leukocytes [11]. Scavenger receptors are known to be multifunctional due to the array of ligands they can recognise, and this has been shown with stabilin-1 [28]. Gathering evidence suggests that this property allows stabilin-1 to contribute to cell adhesion in the low shear flow environments of the lymph nodes and hepatic sinusoids, and therefore could potentially regulate immune cell recruitment to sites of inflammation and tissue injury.

## 3. Stabilin-1 Mediates Lymphocyte Transendothelial Migration across Specialised Vascular Beds

Stabilin-1, also known as FEEL-1 (fasciclin, EGF-like, laminin-type EGF-like, and link domain-containing scavenger receptor-1) [29] or common lymphatic endothelial and vascular endothelial receptor (CLEVER)-1 [30], is a highly evolutionarily conserved type I transmembrane protein and was the first member of the Class H family of scavenger receptor to be described. The expression of stabilin-1 is inducible in conventional vascular endothelia, in response to angiogenic and proinflammatory stimuli [31], but it is constitutively expressed in relatively high levels in the more unconventional non-continuous sinusoidal endothelia of the spleen [32], lymph nodes [33,34] and liver [23]. Additionally, the expression of stabilin-1 appears to be upregulated within the sinusoids of chronically diseased human liver tissues [23]. As a scavenger receptor, stabilin-1 is able to bind a wide variety of ligands, such as modified lipoproteins (LDLs) [35,36], phosphotidylserine expressed on apoptotic cells [37,38,39], secreted protein acidic and rich in cysteine (SPARC) [40], placental lactogen [41] and bacterial microparticles [29]. In addition to its role as a scavenger receptor, there is gathering evidence that stabilin-1 also regulates lymphocyte trafficking across specialised vascular beds.

The adhesive function of stabilin-1 was first described in high endothelial venules (HEVs) and lymphatic vessels, with antibody blockade inhibiting migration of T cells and B cells across HEVs to the draining lymph nodes [30,34]. Consistent with this, our lab subsequently described a role for stabilin-1 in the transendothelial migration of lymphocytes, through the transcellular pathway, in LSEC monolayers in vitro. These studies were undertaken under conditions that mimicked the physiological flow and proinflammatory microenvironment of the hepatic sinusoids during liver injury, and showed that stabilin-1 preferentially mediates the transmigration of regulatory CD4^+^ T cells (T_regs_) and B-cells [19,23,42]. Interestingly, it has previously been suggested that stabilin-1 is also able to support the trafficking of myeloid cells in vivo [30,34]; however, in the context of LSEC, its role in the recruitment of myeloid cell subsets has yet to be explored. The findings that stabilin-1 preferentially mediates the recruitment of T_regs_ and B cells could have significant implications for tumour development in the liver. T_regs_ function as a subset of T cells, which actively suppress effector T cell responses by both cell contact and non-contact pathways [43]. They play an important role in preventing autoimmunity, but have also been implicated in tumour development, with accumulation of T_regs_ in the tumour microenvironment being a poor prognostic feature in several tumours [44,45,46]. More recently, B cells have also been implicated in primary liver cancer development, with IgA producing cells preventing effective CD8 tumour cytotoxic responses in models of hepatocellular cancer (HCC) [47]. We have previously shown that stabilin-1 is not only upregulated at inflammatory sites of leucocyte recruitment, but also in vessels supplying HCCs [23]. In addition, a study undertaken by Karikoski et al. showed significantly fewer T_regs_ in murine tumour models when stabilin-1^−/−^ mice were compared to wild type controls and also demonstrated smaller primary and metastatic tumours in stabilin-1^−/−^ mice, compared to wild type mice [48].

## 4. Stabilin-2

Given its structural homology to stabilin-1, it is perhaps not unexpected that stabilin-2, the second member of the Class H scavenger receptor family, is also found in LSEC and has similarly been implicated in leukocyte recruitment. Stabilin-2, also known as FEEL2 or HARE (hyaluronan receptor for endocytosis), has been shown to bind a diverse range of ligands, such as hyaluronan [49], acLDLs [29], heparin [50,51], apoptotic [52,53], and bacterial microparticles [29]; therefore, it is unsurprising that stabilin-2 is expressed in isolated human LSEC [54] and murine LSEC [55,56]. In addition, stabilin-2 has also been shown to mediate the binding of peripheral blood lymphocytes (PBLs) to human LSEC in vitro [54]. The study, by Jung et al., demonstrated that α_M_β_2_ integrin on PBLs was able to bind to the fasciclin 1 (FAS1) domains of stabilin-2, mediating the firm adhesion step of the adhesion cascade under flow conditions in vitro. Nevertheless, this still remains the only investigation of lymphocyte binding to LSEC-expressed stabilin-2 to date and, as monocytes [57] and neutrophils [58] also express α_M_β_2_, it would be interesting to investigate whether or not stabilin-2 is also able to mediate the binding of these myeloid populations. Furthermore, to the authors’ knowledge, the expression of stabilin-2 has not yet been explored in chronically inflamed human liver tissues or experimental inflammatory models, and so the physiological relevance of stabilin-2’s ability to bind leukocytes is also unknown.

## 5. The Role of Macrophages in Liver Disease

Whilst lymphocyte infiltration is a hallmark of all chronic adult liver diseases and the role of endothelial/lymphocyte interaction is a key step in this process, there is now a significant body of evidence that macrophages also play a major role in orchestrating liver injury. Macrophages are a critical arm of the innate immune system, acting as sentinels to detect foreign pathogens and danger signals released by tissue injury. They are armed with a range of pattern recognition receptors that include both toll-like receptors (TLRs) and scavenger receptors [59]. In the liver, the tissue resident macrophages, known as Kupffer cells, positioned within the hepatic sinusoids, are relatively stationary and do not migrate. Recent studies have demonstrated that the source of tissue-resident macrophages, such as Kupffer cells, have an origin independent from circulating monocytes and the adult bone marrow haematopoiesis, and are derived from the fetal yolk sac and are long-lived and self renew [60]. With the onset of liver injury due to hepatocyte damage from toxins or pathogens, the release of danger signals leads to the activation of Kupffer cells through recognition by surface receptors and the downstream triggering of the inflammasome [61]. This leads to the release of factors such as the chemokine CCL2, which play a critical role in the recruitment of circulating monocyte populations into liver tissue during injury, which then mature into distinct macrophage subsets [62,63]. Careful analysis of these macrophage populations in a murine model of acute liver injury induced by administration of N-acetyl-p-aminophenol (APAP) demonstrates a reduction in the resident Kupffer cell population [64]. The injury triggered a significant infiltration of monocytes from the circulation, which were characterised by Ly6C high expression, and this process was mediated by the chemokine receptor CCR2 on these monocytes. A third subset was identified characterised by Ly6C low expression and predominated during the resolving phase. The Ly6C low subset of macrophages are known to have a patrolling behaviour and have higher expression of scavenger receptors, with the spleen acting as a reservoir for these monocytes whilst the Ly6C high population being derived from the bone marrow [65]. Ly6C high monocytes in mice are analogous to human classical (CD14^++^/CD16^-^) and intermediate monocytes (CD14^+^CD16^+^), with Ly6C low monocytes representative of the non-classical monocytes with higher CD16 expression (CD14^+^CD16^++^). Intriguingly, during sterile injury of the liver a further subset of macrophages were noted, which infiltrated from the peritoneal cavity through the liver capsule and were characterised by the transcription factor GATA-6 [66]. Along with these phenotypic subsets, macrophages are profoundly sensitive to their microenvironment, leading to plasticity and the suggestion that they can switch from one phenotype to the other. Building on these phenotypic studies and going on to use macrophage depletion techniques at different stages of injury, it has become clear that macrophages can have opposing roles at different stages of liver injury. During the initiation of damage, infiltrating macrophages contribute to pro-inflammatory pathways and the progression of liver fibrosis, and inhibiting their recruitment or depleting them has been shown to prevent injury progression. However, their depletion once the injury is removed has been shown to delay resolution and, in chronic models, prevent tissue remodeling [67]. These macrophage subsets are characterised by distinct surface receptors, including scavenger receptors as well as characteristic secretory profiles of cytokines and mediators of tissue remodelling that drive the functional impact on inflammation and resolution in the liver [68]. The mechanism of how macrophage subsets influence inflammation and fibrosis is of great interest in order to understand their potential regarding cell therapy and the potential of manipulating macrophage behaviour in situ [69]; there is now a strong body of evidence that macrophages contribute mediators that can activate hepatic stellate cells (HSCs). HSCs, as mentioned earlier, are the liver resident pericyte and are characterised by neural markers (glial fibrillary acidic protein (GFAP) and synaptophysin), store Vitamin A [70], and play a central role in chronic liver disease. Upon liver injury, they become activated and transform into myofibroblasts, which subsequently promote tissue loss and architectural distortion through the excessive release of extracellular matrix (ECM) and increased contractility. Macrophages are known to release transforming growth factor beta (TGF-β), a key activator of HSCs, as well as chemokines, which promote HSC migration and other proinflammatory cytokines that drive fibroblast activation [71]. With better understanding of long-term outcomes in patients with liver disease, and the use of animal models, it is clear that liver fibrosis does not follow a linear progressive pathway but is a highly dynamic process of repair and damage, with dramatic resolution of fibrosis possible [72]. The mechanisms of how macrophages contribute to this repair is also being elucidated, predominantly using rat and murine models of repetitive toxin-induced injury that lead to HSC activation and hepatic fibrosis, such as carbon tetrachloride (CCl_4_), followed by careful analysis of the healing phase. Matrix metalloproteinases (MMPs) play a key role in the degradation of ECM, and pro-resolution macrophages have been shown to express specific MMPs including MMP12 and MMP13 [73]. Interestingly, the physical process of phagocytosis of apoptotic cells by macrophages drives a phenotypical change in macrophages, leading to the down-regulation of fibrotic/inflammatory factors to the upregulation of proresolution factors including MMPs but also arginase-1, which has been shown to have anti-fibrotic properties [68].

## 6. Stabilin-1 Plays a Distinct Role in Macrophage-Mediated Tissue Remodelling during Liver Injury

It has long been recognised that stabilin-1 is expressed on specific populations of tissue-resident macrophages, including placental macrophages, subpopulations of skin macrophages and lymph node macrophages [31,32,74,75,76]. Interestingly, previous studies of the normal liver have not described it on Kupffer cells and demonstrated expression restricted to the liver sinusoidal endothelium [74]. Stabilin-1 appears to have a relatively low expression on circulating immature monocytes, but has been shown to be upregulated in the setting of familial hypercholesterolaemia (FH) [77]. It was hypothesised that stabilin-1 was induced on these monocytes in order to support the clearance of increased deposits of oxidised low density lipoproteins (oxLDLs) from vessel walls associated with FH. In vitro studies with monocytes have clearly shown that stabilin-1 is highly upregulated by stimulation with IL-4 and dexamethasone [49]. In these macrophages, stabilin-1 has been shown to have both scavenging roles and contributes to intracellular sorting. For example, efficient uptake of SPARC by alternatively activated macrophages was mediated by stabilin-1 [40]; in addition, stabilin-1 has been shown to interact with human Glyco_18-domain-containing proteins and regulates their trafficking to lysosomes [78]. These findings have led to the conclusion that stabilin-1 makes an important functional contribution to alternatively-activated (M2) macrophages, as opposed to classically-activated (M1) macrophages, which are generated in cell culture by stimulating monocytes with interferon-γ. M2 macrophages have traditionally been considered anti-inflammatory macrophages, with features that promote wound healing and resolution but are also features of tumour associated macrophages (TAMs). Recent work has highlighted stabilin-1 upregulaton in TAMs in a range of cancers, and in an in vivo model of breast cancer the uptake of SPARC, a tumour inhibiting factor, by stabilin-1 on TAMs promoted tumor progression [79].

It is now clear that, whilst this M1/M2 polarisation can be generated in vitro, the presence of such polarized macrophage subsets in the disease setting is questionable. Phenotypic profiles of macrophages in disease settings appear to be much more dynamic than the M1/M2 paradigm, and this has been confirmed in the context of the liver, where macrophages isolated from hepatic tissue during injury expressed inflammatory and resolution markers simultaneously, suggesting alternative classification is required [80]. In murine models of liver injury, CD11b+/F4/80+ macrophage subsets that were defined by Gr1 expression and lacked expression of neutrophil marker Ly6G were shown to be proinflammatory and promote fibrosis, whereas the low expression of Ly6C identified subsets that were anti-inflammatory and contributed to healing [63,68]. Studies in human liver disease identified the accumulation of the CD14++CD16+ subsets of macrophages, which expressed pro-inflammatory and profibrogenic cytokines and are therefore likely to play an important role in liver fibrosis [81].

**Figure 4:**
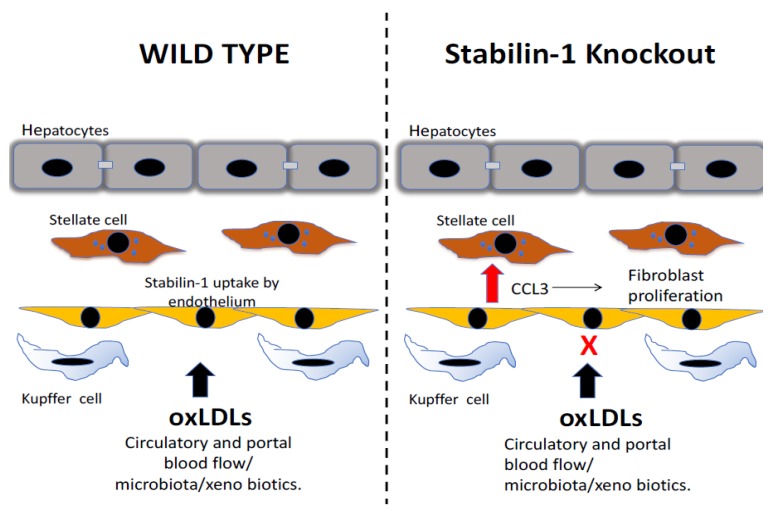
The scavenging role of stabilin maintains homeostasis in the liver by the uptake of products of oxidative stress. Stabilin-1 contributes to the hepatic uptake of circulating oxidized low-density lipoproteins (oxLDLs). In the setting of stabilin-1 deficiency, these oxLDLs lead to a proinflammatory response specifically, leading to increased levels of the chemokine CCL3, which drives the proliferation of liver-resident fibroblasts and causes an increase in collagen fibres in the livers of stabilin-1 knockout mice.

**Figure 5:**
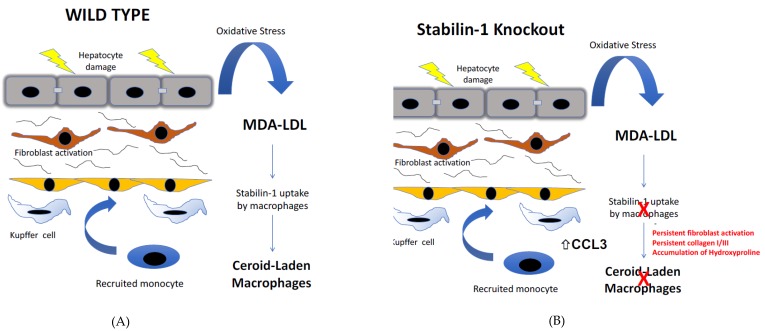
Stabilin-1 expression on hepatic macrophages protects against excessive tissue damage from chronic oxidative stress. (**A**) In models of chronic liver injury, the repetitive damage to hepatocytes leads to oxidative stress and lipid peroxidation, which leads to the formation of malondialdheyde-lipoproteins (MDA-LDL), which accumulate in the liver. Stabilin-1 expression on hepatic macrophages leads to the uptake of MDA-LDL, which leads to the formation of ceroid-laden macrophages that are found at sites of scarring. The active uptake of MDA-LDL by stabilin-1 positive macrophages suppresses the release of pro inflammatory mediators such as CCL3. (**B**) In the setting of stabilin-1 deficiency, there is a loss of these ceroid-laden macrophages and a lack of accumulation of MDA-LDL within hepatic macrophages. The stabilin-1-deficient hepatic macrophages are shifted to a pro-inflammatory phenotype including excessive release of CCL3, and this is associated with excessive scarring from activated liver fibroblasts and delayed healing after liver injury.

The generation of stabilin-1 knockout mice has helped to elucidate the contribution of stabilin-1 to hepatic macrophage function. Stabilin-1 knockout mice demonstrated that these mice had a normal lifespan, but histological analysis of the liver demonstrated a mild deposition of collagen fibres, which were absent from wild type counterparts; however, combined knockout of stabilin-1 and stabilin-2 led to premature death. This increased mortality rate was attributed to glomerular fibrosis, driven by diminished scavenging of profibrotic factors, with growth differentiation factor(GDF)-15 particularly implicated [56]. In our studies, utilising stabilin-1 knockout mice, we aimed to understand the contribution of stabilin-1 in the context of liver injury and repair. Analysis of well-established models of chronic liver injury, including CCl_4_ repetitive injury and a resolution phase, revealed stabilin-1 deficiency had a significant impact on both fibrosis deposition within the liver and its subsequent resolution. Stabilin-1-deficient mice developed an exacerbation of fibrosis in-keeping with the fibrotic changes noted in previous findings, but also a profound impairment of fibrosis resolution. Histological analysis demonstrated that whilst stabilin-1 was restricted to endothelium in the uninjured liver, upon chronic damage, there was a sub-population of stabilin-1^+^ macrophages detectable. Mechanistically, the recognition of oxLDLs, specifically malondialdehyde-LDL, by stabilin-1 macrophages led to a suppression of the production of the pro-inflammatory chemokine CCL3, which is known to have a significant effect on fibroblast phenotype. The increased expression of CCL3 from macrophages led to high numbers of GFAP^+^ fibroblasts, which would explain the excessive deposition of ECM (Figure 4 and Figure 5). Interestingly, the deficiency of stabilin-1 had transcriptional effects on liver-derived macrophages, skewing them to a pro-inflammatory phenotype with increased TNF-alpha expression and reduced arginase levels. In addition, the suppression of Ly6C low populations, whose increase in injury, as described earlier, is critical to fibrosis resolution, was evident [35]. The importance of stabilin-1 expression on macrophages in fibrosis resolution was confirmed by the use of both cell-specific knockouts and the adoptive transfer of macrophages to reverse this defect in the setting of stabilin-1 deficiency during fibrosis resolution [35]. Additional transcriptional analysis of hepatic macrophages demonstrated higher expression on mature macrophages rather than immature recruited monocytes. Whether the major contribution of macrophage stabilin-1 in liver fibrosis is due to upregulation on resident Kupffer cells or monocyte- derived macrophages that are recruited from the circulation is still unclear, as experiments with organ-specific knockout or Kupffer cell-specific knockout of stabilin-1, to our knowledge, are yet to be performed.

## 7. Conclusions

Chronic liver disease is a major global cause of mortality and death, and currently there are no licensed therapies to treat the tissue fibrosis that drives these conditions. Gaining a better understanding of the regulatory pathways in disease pathophysiology could lead to novel therapies. In this review, we have highlighted the emerging contribution of the scavenger receptor stabilin-1 to two key pathogenic mechanisms in progressive liver disease, namely, the recruitment of lymphocytes and the scavenging function of macrophages. Stabilin-1 is expressed at sites of lymphocyte recruitment in the human liver, including the hepatic sinusoidal channels and on neovessels at site of liver tissue injury. It is also upregulated on hepatic macrophages during disease, and actively regulates their release of pro-inflammatory mediators. Scavenger receptors have been traditionally considered to be a family of receptors with significant redundancy, but this assumption is being challenged, and they are now considered to be an important link between tissue injury and immune responses [82]. Stabilin-1 is an example of a homeostatic receptor within the liver, but also has an additional impact on the immune microenvironment during liver inflammation and fibrosis.
